# Gut Microbiota and Intestinal Epithelial Myd88 Signaling Are Crucial for Renal Injury in UUO Mice

**DOI:** 10.3389/fimmu.2020.578623

**Published:** 2020-12-22

**Authors:** Ingrid Kazue Mizuno Watanabe, Magaiver Andrade-Silva, Orestes Foresto-Neto, Raphael José Ferreira Felizardo, Marco Aurélio Costa Matheus, Reinaldo Correa Silva, Marcos Antônio Cenedeze, Tâmisa Seeko Bandeira Honda, Luiz Augusto Buoro Perandini, Rildo Aparecido Volpini, Alvaro Pacheco-Silva, Niels Olsen Saraiva Câmara

**Affiliations:** ^1^ Department of Immunology, Institute of Biomedical Science, University of São Paulo, São Paulo, Brazil; ^2^ Nephrology Division, Department of Medicine, Federal University of São Paulo, São Paulo, Brazil; ^3^ Department of Nephrology, University of São Paulo, School of Medicine, São Paulo, Brazil; ^4^ Kidney Transplant Unit, Hospital Israelita Albert Einstein, São Paulo, Brazil

**Keywords:** chronic kidney disease, gut microbiota, Myd88, inflammation, fibrosis

## Abstract

Increasing evidence shows the essential participation of gut microbiota in human health and diseases by shaping local and systemic immunity. Despite an accumulating body of studies showing that chronic kidney disease (CKD) is closely associated with disturbances in the composition of gut microbiota, it remains unclear the importance of gut microbiota in the onset and development of CKD. For the purpose of untangling the role of gut microbiota in CKD, gut microbiota was depleted with a pool of broad-spectrum antibiotics in mice submitted to unilateral ureteral obstruction (UUO). Depletion of gut microbiota significantly decreased levels of proinflammatory cytokines and fibrosis markers, attenuating renal injury. Additionally, to study whether the pathogenic role of gut microbiota is dependent of microbial-host crosstalk, we generated mice lacking Myd88 (myeloid differentiation primary response gene 8) expression in intestinal epithelial cells (IECs) and performed UUO. The absence of Myd88 in IECs prevented a bacterial burden in mesenteric lymph nodes as observed in WT mice after UUO and led to lower expression of proinflammatory cytokines and chemokines, reducing deposition of type I collagen and, ultimately, attenuating renal damage. Therefore, our results suggest that the presence of gut microbiota is crucial for the development of CKD and may be dependent of Myd88 signaling in IECs, which appears to be essential to maturation of immune cells intimately involved in aggravation of inflammatory scenarios.

## Introduction

Chronic kidney disease (CKD) has been considered as a global public health problem. In 2017, CKD was ranked as the 12th leading cause of death and the number of cases reached almost 700 million worldwide (1). The onset and progression of CKD involve a complex interplay among metabolic, neuroendocrine and immunologic events, which generates a high risk clinical phenotype (2). In the past decades, other players have been included in this scenario, including the gut and its indigenous residents so-called the gut microbiota.

Despite the existence of a large variety of causative factors in the onset and progression of CKD, accumulating evidence has continuously linked local and systemic outcomes from kidney damage to dysbiosis. Kidney failure provokes accumulation of uremic toxins, which, in turn, can impair the integrity of intestinal barrier and disturb the composition and functionality of gut microbiota (dysbiosis) enhancing toxin production and inflammation in CKD. Accumulation of toxins originated from the gut microbiota in CKD, such as p-cresol sulfate and TMAO, has been linked to the progression of kidney function (3, 4). Profound disturbances in gut microbiota may also alter the levels of microbial metabolites with benefits to the host metabolism, such as short-chain fatty acids (SCFAs), which participate in the modulation of inflammatory responses in models of kidney injury (5–7).

Alterations in the microbial composition and functionality can influence the maintenance of intestinal barrier. In homeostatic conditions, intestinal epithelial cells (IECs) and immune cells “feel and react” to microbial stimuli orchestrating a symphony of immune responses and reinforcing the intestinal barrier to keep a balance between tolerance and pathogenic immunity. The integrity of intestinal barrier critically relies on the fitness of IECs, which are central players of a microbial-dependent strategy for intestinal repair and homeostasis. IECs sense microbial-associated molecular patterns (MAMPs), through pattern recognition receptors (PRRs), including Toll-like receptors (TLRs) and NOD-like receptors (NLRs), promoting proliferation and differentiation of epithelial cells, reinforcement of tight junctions (8), and secretion of IgA and antimicrobial peptides (AMPs) in the lumen (9).

The dysfunction of intestinal barrier has been observed in a variety of diseases, including type 1 diabetes mellitus, inflammatory bowel disease and coeliac disease (10). In the context of CKD, uremia has been strongly associated with disturbances in tight junction dynamics. Experimental studies observed a pronounced depletion of components of intestinal epithelial tight junction in uremic animals (11). Moreover, Varizi et al. observed that *in vitro* treatment with urea remarkably reduced the expression of claudin-1, occludin and ZO-1 in IECs, which was accompanied by lower transepithelial electrical resistance (12). Impaired intestinal barrier not only aggravates local inflammation but could also contribute to systemic inflammation. Indeed, circulating endotoxemia and bacterial-derived DNA fragments were found in CKD patients (13, 14). Furthermore, the blood levels of endotoxins seem to be correlated with CKD stage (14).

Together, evidence points to the importance of gut microbiota and intestinal barrier fitness in the pathogenesis of CKD. Therefore, the purpose of this study was to evaluate the role of gut microbiota in the onset and development of CKD and whether the influence of gut microbiota was dependent of the microbial-host crosstalk, specifically of the Myd88 signaling in IECs.

## Material and Methods

### Mice

All animal procedures described in this study were approved by the Ethics Committee in Animal Research of the Federal University of São Paulo (Protocol number: 7562120416) and all the animal study methods were performed according to the Ethical Principles in Animal Research. Animals were held under temperature-controlled conditions on a 12-h light cycle with access to food and water *ad libitum*.

Gut microbiota was depleted in 6-week-old C57Bl/6 mice by oral administration of broad-spectrum antibiotics (ABX) [1 g/L ampicillin, 1 g/L metronidazole, 1 g/L neomycin and 0.5 g/L vancomycin (Sigma-Aldrich)] *via* autoclaved drinking water with 1% (wt/vol) glucose for 4 weeks, as previously described (15). During ABX treatment, bacterial load in the feces was assessed once in a week. Briefly, fresh fecal pellets were resuspended in sterile PBS, and an aliquot of the suspension was inoculated in 5 ml of Luria-Bertani (LB) broth and kept at 37°C for 3 days. Bacteria concentration in the culture was estimated through the optical density at 600 nm. After 3 weeks of ABX treatment, irreversible unilateral ureteral obstruction (UUO) was performed in control and ABX mice, as previously described (16).

Selective deletion of Myd88 in IECs (Myd88^ΔIEC^) was obtained through the intercross of B6.129P2-MyD88 tm1Defr/J (Myd88^fl/fl^) and B6N.Cg-Tg (Vil1-Cre)997Gum/J strains. After confirmation of Myd88 deletion in isolated IECs, mice were submitted to UUO. After 7 days of UUO, mice were euthanized, and samples [blood, colon, liver, mesenteric lymph node (MLN) and kidney] were collected for further analysis.

### Pelvic Biochemical Urinary Parameters

Total protein and creatinine in urine were quantified using commercial kits according to instructions (Labtest). Urinary albumin content was estimated using dye-binding technique. Briefly, under reducing conditions, urine samples were resolved by 10% SDS-polyacrylamide gel electrophoresis. Gels were stained with 0.1% Coomassie blue R-250 solution for 2 h and destained overnight. Albumin concentration was determined according to the intensity of the bands using a positive loading control (bovine serum albumin, 0.2 mg/ml) using ImageJ software.

### Cytokines Quantification

Serum levels of IL-6 were quantified using available commercial kit (R&D Systems) according to manufacturer’s instructions. Quantification of cytokines and chemokines in kidney and colon samples were performed using Bio-Plex Pro mouse cytokine, chemokine, and growth factor assay (Bio-Rad) according to manufacturer’s instructions. Briefly, aliquots of tissue homogenate were incubated with premixed “color-coded” magnetic beads in a shaker at room temperature for 30 min. After washing steps, incubation with biotinylated detection antibody and streptavidin PE conjugate were performed subsequently, allowing measurement of a specific cytokine later in the laser detection step. Cytokine concentrations were determined using standards curves and results were expressed as pg/mg of total protein.

### Immunohistochemistry

Formalin-fixed tissue was embedded in paraffin using standard procedure and histological sections were immunostained by standard avidin-biotin-peroxidase methodology (Vectastain ABC Kit, Vector Laboratories) using diaminobenzidine (DAB) as the chromogen. Profibrotic and cell proliferation status were assessed using antibodies anti-type I collagen (1:200, Abcam), anti-fibroblast-specific protein 1 (FSP-1) (1:800, Dako) and anti-KI-67 (1:1,000, Abcam), respectively. Digital microphotographs of renal cortex were obtained at 40-fold magnification using a Carl Zeiss Axioskop 40 microscope (Carl Zeiss Microscopy) and AxioCam MRc5 digital camera (Carl Zeiss Microscopy). The area stained positive for type I collagen, FSP-1 and KI-67 was quantified as percentage of total cross-sectional area using Axiovision software (Carl Zeiss Microscopy).

### Fluorescence *In Situ* Hybridization

Segments of distal colon containing feces were harvested and fixed in methacarn solution at 4°C for 24 h and embedded in paraffin using standard procedure. Briefly, deparaffinized sections were incubated with Eubacteria probe (AF488-conjugated, Sigma-Aldrich) in a humid chamber at 50°C overnight. After washing step, sections were incubated with lectin from *Ulex europaeus* (TRITC-conjugated, Sigma-Aldrich) at room temperature for 2 h. After mounting step with Vectashield (Vector laboratories), images were obtained using confocal microscope at a magnification of 40×.

### Detection of Bacterial DNA in Organ Samples

DNA from MLN and liver samples (collected in sterile conditions) was extracted using DNeasy Blood & Tissue Kit (Qiagen) according to manufacturer’s instructions. All reactions were carried out with 20 ng of DNA through Sybr system using primers designed for bacterial 16S gene and for mouse genomic DNA (**Table 1**). Quantification of bacterial 16S rDNA was normalized using mouse genomic DNA (pIgR genomic region) as internal control and calculated using the ΔΔCT-method. Results were expressed as an n-fold difference in relation to the expression of matched controls.

**Table 1:** Primer sequences used for qPCR.

Gene	Forward	Reverse
*IL-1b*	GCC ACC TTT TGA CAG TGA TGA	TGA TGT GCT GCT GCG AGA TT
*IL-4*	CCC CAG CTA GTT GTC ATC CTG	CAA GTG ATT TTT GTC GCA TCC G
*IL-6*	TCT CTG CAA GAG ACT TCC ATC C	AGA CAG GTC TGT TGG GAG TG
*IL-12a*	AGA CAT CAC ACG GGA CCA AAC	CCA GGC AAC TCT CGT TCT TGT
*TNF-a*	GCC CCC AGT CTG TAT CCT TCT AA	ACT GTC CCA GCA TCT TGT GTT TC
*Csf2* (GM-CSF)	AGG GTC TAC GGG GCA ATT TC	GGC AGT ATG TCT GGT AGT AGC TG
*Ccl4* (MIP-1b)	TTC CTG CTG TTT CTC TTA CAC CT	CTG TCT GCC TCT TTT GGT CAG
*Col1a1*	CGT ATC ACC AAA CTC AGA AG	GAA GCA AAG TTT CCT CCA AG
*Fibronectin*	TAC AAC AAC CGG AAT TAC AC	GAT ACA TGA CCC CTT CAT TG
HPRT	CTC ATG GAC TGA TTA TGG ACA GGA C	GCA GGT CAG CAA AGA ACT TAT AGC C
*GAPDH*	AGG TCG GTG TGA ACG GAT TTG	TGT AGA CCA TGT AGT TGA GGT CA
*pIgR* genomic region	TTT GCT CCT GGG CCT CCA AGT T	AGC CCG TGA CTG CCA CAA ATC A
*16S rDNA* (bacteria)	TGG CTC AGG ACG AAC GCT GGC GGC	CCT ACT GCT GCC TCC CGT AGG AGT

### Gene Expression

Total RNA from colon and kidney samples were extracted with Trizol^®^ (Invitrogen), and cDNA was synthesized using High-Capacity cDNA Reverse Transcription Kit (Applied Biosystems). Gene expression of proinflammatory cytokines and profibrotic markers were performed based on Sybr system using designed primers listed in **Table 1**. Quantification of target gene expression was normalized using HPRT or GAPDH as internal controls and calculated using the ΔΔCT-method. Results were expressed as an n-fold difference in relation to the expression of matched controls. Analyses were performed using QuantStudio™ Design & Analysis Software v1.5.0 (Applied Biosystems).

### Western Blotting

Kidney samples were homogenized in ice-cold RIPA buffer containing protease inhibitors cocktail (Roche Applied Science). Aliquots of 20 µg of protein were subjected to electrophoresis under reducing conditions. Proteins transferred to nitrocellulose membranes were probed overnight with mouse monoclonal anti-α smooth muscle actin (α-SMA) antibody (1:1,000, Dako), and, sequentially, incubated with appropriate secondary antibody. The bands were detected by chemiluminescence (Clarity Western ECL Substrate, Bio-Rad) according to the manufacturers’ recommendations. Results were normalized relative to α-tubulin expression.

### Statistics

Results were expressed as mean ± SE. Student *t* test was performed to compare two groups, and one-way ANOVA with Tukey’s *post hoc* test was performed to compare more than three groups using GraphPad Prism 5.0 (GraphPad Software). Statistical significance was set at *p* ≤ 0.05.

## Results

### Depletion of Gut Microbiota Protects Against UUO Injury

**Figure 1:**
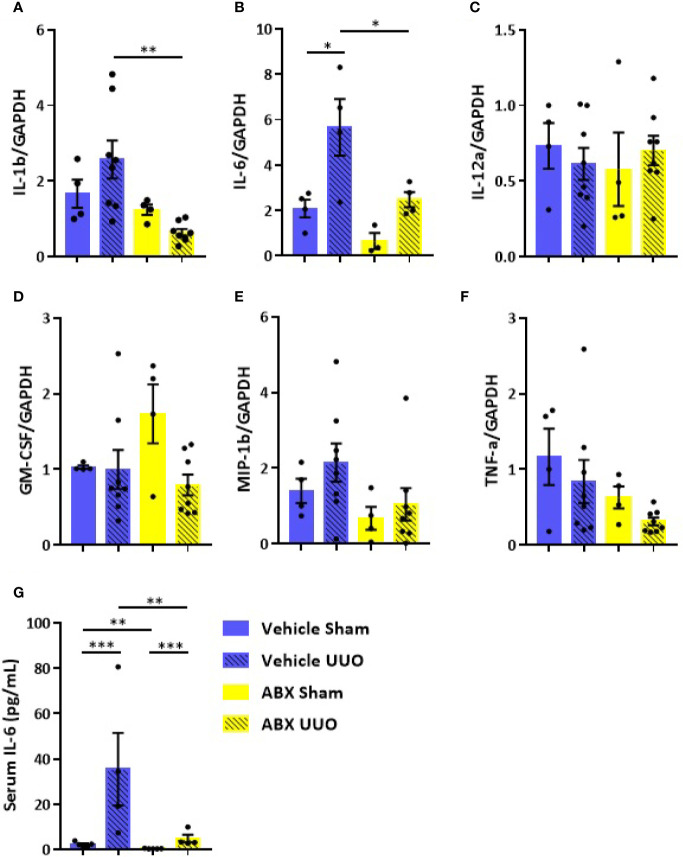
Impact of depleted gut microbiota on the colon and systemic inflammation in CKD. Gene expression of **(A)** IL-1b, **(B)** IL-6, **(C)** IL-12a, **(D)** GM-CSF, **(E)** MIP-1β, and **(F)** TNF-α in the colon of Vehicle and ABX-treated mice submitted or not to UUO (N = 4 to 8). **(G)** Serum levels of IL-6 of Vehicle and ABX-treated mice submitted or not to UUO (N = 4). *p < 0.05; **p < 0.01; ***p < 0.001.

Treatment with ABX was effective in lowering gut bacterial load and in modulating gene expression of some components of intestinal barrier (**Figure S1**). Depletion of gut microbiota was followed by upregulation of occludin and ZO-1, which are considered key elements of tight junctions. CKD has been implicated in disruption of intestinal epithelial tight junctions, which has been associated with local and systemic inflammation (**Figure S1**). UUO led to increased gene expression of proinflammatory cytokines, such as IL-1b and IL-6, in the colon (**Figure 1**), which was prevented by depletion of gut microbiota. Systemic inflammatory status was also greatly influenced by UUO, as indicated by a 7-fold increase of serum levels of IL-6 in untreated group (**Figure 1G**), which was partially prevented by ABX treatment.

**Figure 2:**
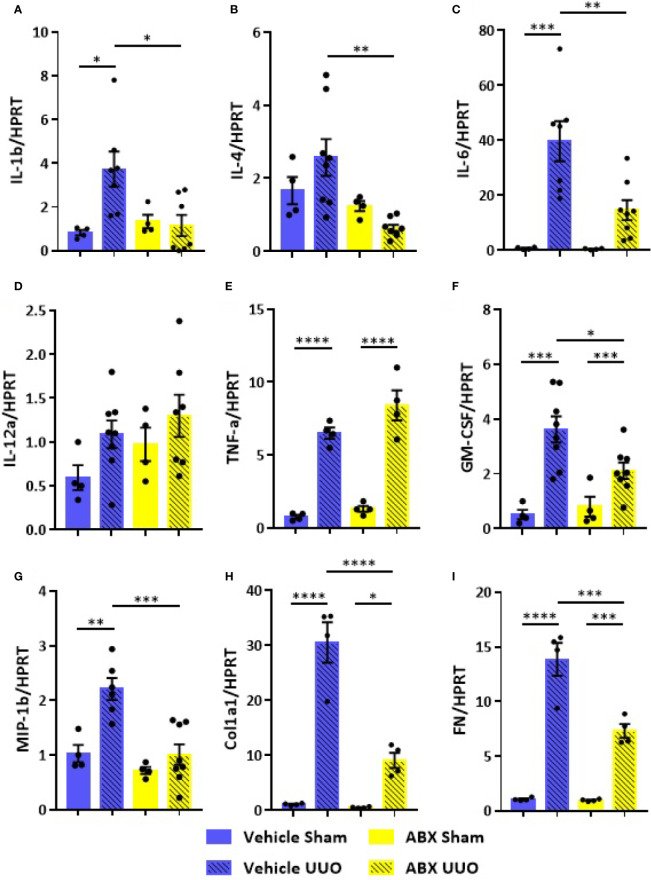
Impact of depleted gut microbiota on the kidney in CKD. Gene expression of **(A)** IL-1b, **(B)** IL-4, **(C)** IL-6, **(D)** IL-12a, **(E)** TNF-α, **(F)** GM-CSF, **(G)** MIP-1β, **(H)** Col1a1, and **(I)** fibronectin (FN) in the kidney of Vehicle and ABX-treated mice submitted or not to UUO (N = 4 to 8). *p < 0.05; **p < 0.01; ***p < 0.001; ****p < 0.0001.

**Figure 3:**
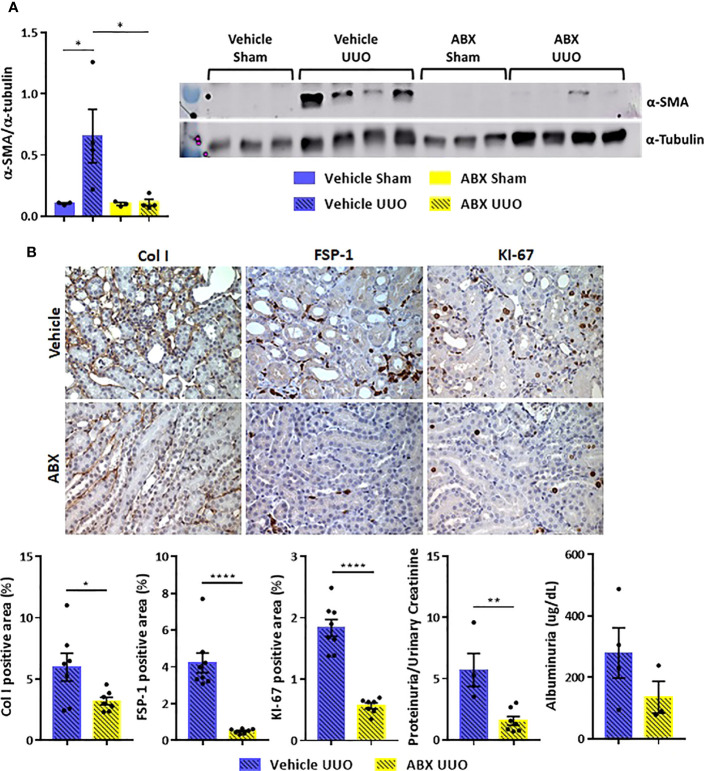
**(A)** Protein expression of α-SMA in the kidney of Vehicle and ABX-treated mice submitted or not to UUO (N = 3 to 4) by Western blotting. **(B)** Representative images and positive area (%) of immunohistochemical staining for type I collagen (Col I), FSP-1 and KI-67 in the kidney of Vehicle and ABX-treated mice submitted to UUO (N = 7 to 8); concentration of total protein normalized by creatinine in pelvic urine (N = 4 to 7); estimation of albuminuria in pelvic urine collected from Vehicle and ABX-treated mice submitted to UUO (N = 3 to 4). *p < 0.05; **p < 0.01; ****p < 0.0001.

The systemic inflammation observed after UUO was followed by increased gene expression of proinflammatory cytokines in the kidney of untreated mice. On the other hand, ABX-treated group showed strikingly lower expression of cytokines, such as IL-1b, IL-4, and IL-6, and chemokines (GM-CSF and MIP-1β) after UUO (**Figure 2**). In addition to that, depletion of gut microbiota modulated the expression of markers of tubulointerstitial fibrosis. Decreased gene expression of profibrotic markers, such as fibronectin and type I collagen, was observed in the kidney of ABX-treated mice (**Figures 2H, I**). We also observed lower protein expression of indicators of kidney injury, including α-SMA and type I collagen (**Figure 3**). In addition, depletion of gut microbiota affected the expression of markers of cell activation, such as FSP-1 and KI-67. ABX treatment led to lower quantity of FSP-1 positive cells in the renal intestitium, and decreased number of KI-67 positive cells, including renal tubular cells and interstitial cell populations (**Figure 3B**). This protection against UUO injury was followed by better pelvic urinary parameters. Abx-treated mice presented lower levels of protein in pelvic urine after UUO compared with untreated group (**Figure 3B**). Taken together, our results indicated that the proinflammatory and profibrotic scenarios caused by UUO are dependent of the presence of gut microbiota.

### Myd88 Signaling in IECs Participates in UUO Injury

**Figure 4:**
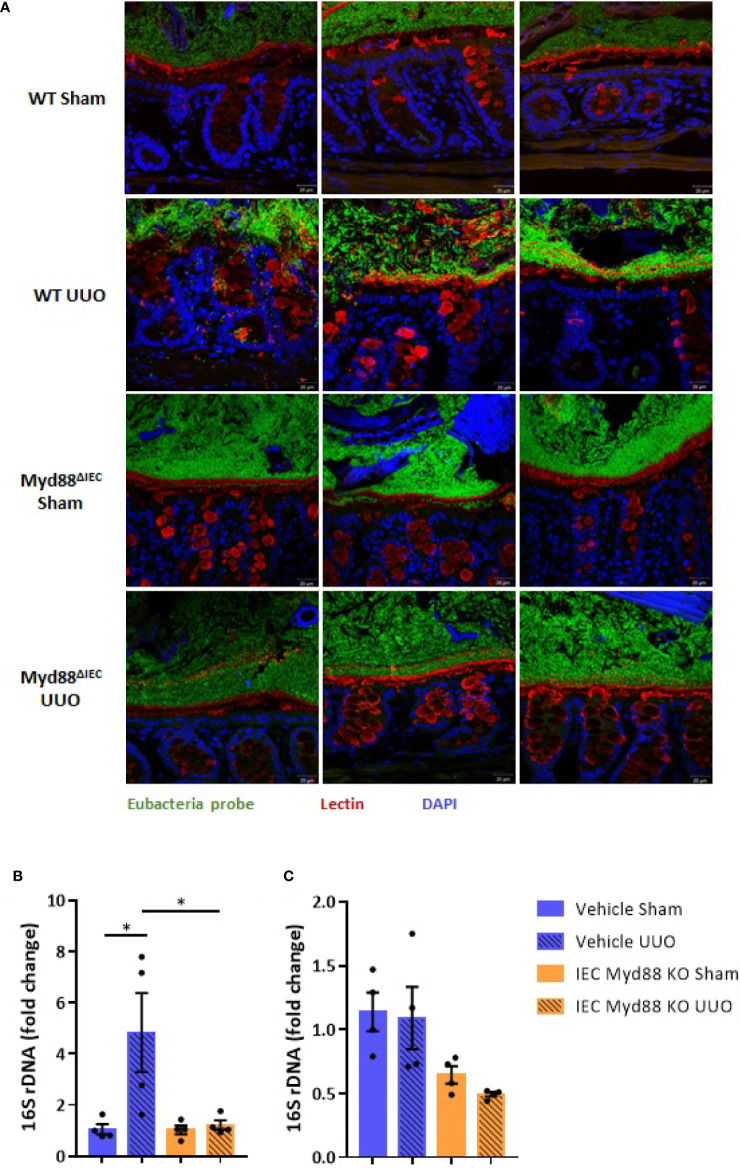
**(A)** Fluorescence *in situ* hybridization (FISH) analysis of the total bacteria in the colon. Visualization of colon fragments containing feces labeled with probe for Eubacteria (green) and FITC-conjugated lectin (red) for mucus (x40 fold magnification). **(B)** Quantification of bacterial DNA in the mesenteric lymph node (MLN) of untreated wild type (WT) and Myd88^ΔIEC^ mice submitted or not to UUO based on gene expression of 16S rDNA (N = 4). **(C)** Quantification of bacterial DNA in the liver of untreated wild type (WT) and Myd88^ΔIEC^ mice submitted or not to UUO based on gene expression of 16S rDNA (N = 4). *p < 0.05.

**Figure 5:**
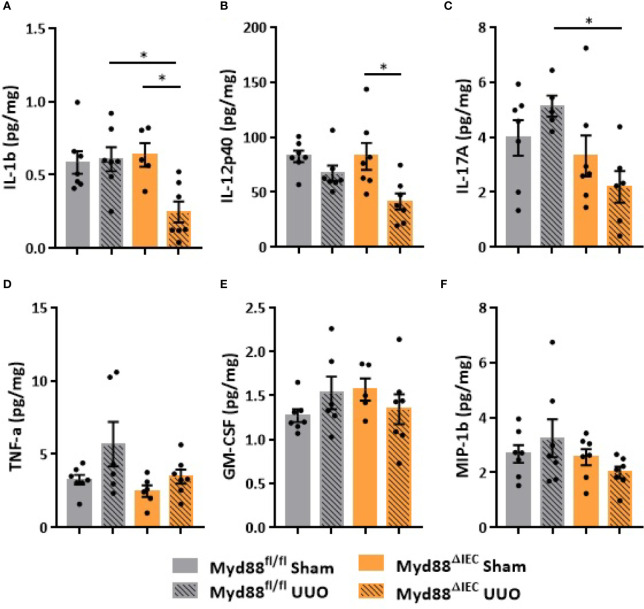
Effect of Myd88 signaling in IECs on the colon in CKD. Concentration of **(A)** IL-1b, **(B)** IL-12p40, **(C)** IL-17A, **(D)** TNF-α, **(E)** GM-CSF, and **(F)** MIP-1β in the colon of Myd88^fl/fl^ and Myd88^ΔIEC^ mice submitted or not to UUO determined by multiplex cytokine assay (N = 5 to 7). *p < 0.05.

**Figure 6:**
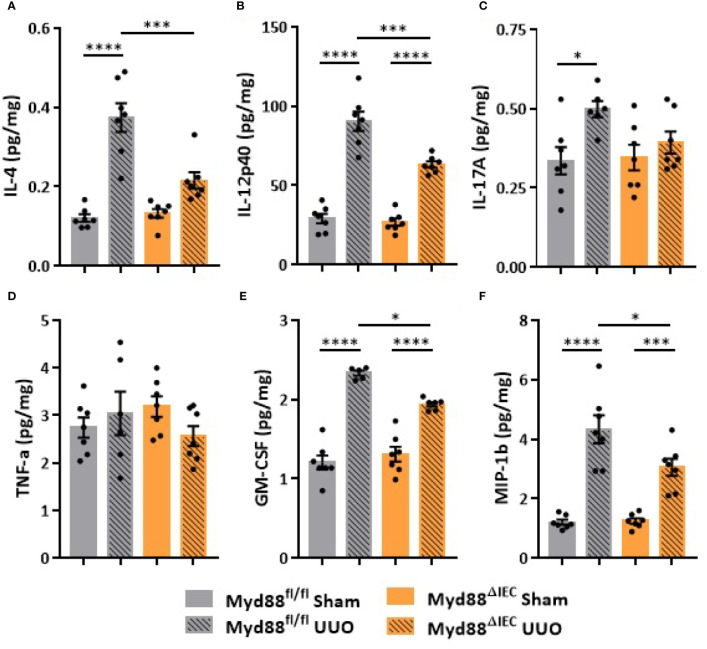
Effect of Myd88 signaling in IECs on the inflammatory profile of the kidney in CKD. Concentration of **(A)** IL-4, **(B)** IL-12p40, **(C)** IL-17A, **(D)** TNF-α, **(E)** GM-CSF, and **(F)** MIP-1β in the kidney of Myd88^fl/fl^ and Myd88^ΔIEC^ mice submitted or not to UUO determined by multiplex cytokine assay (N = 5 to 7). *p < 0.05; ***p < 0.001; ****p < 0.0001.

**Figure 7:**
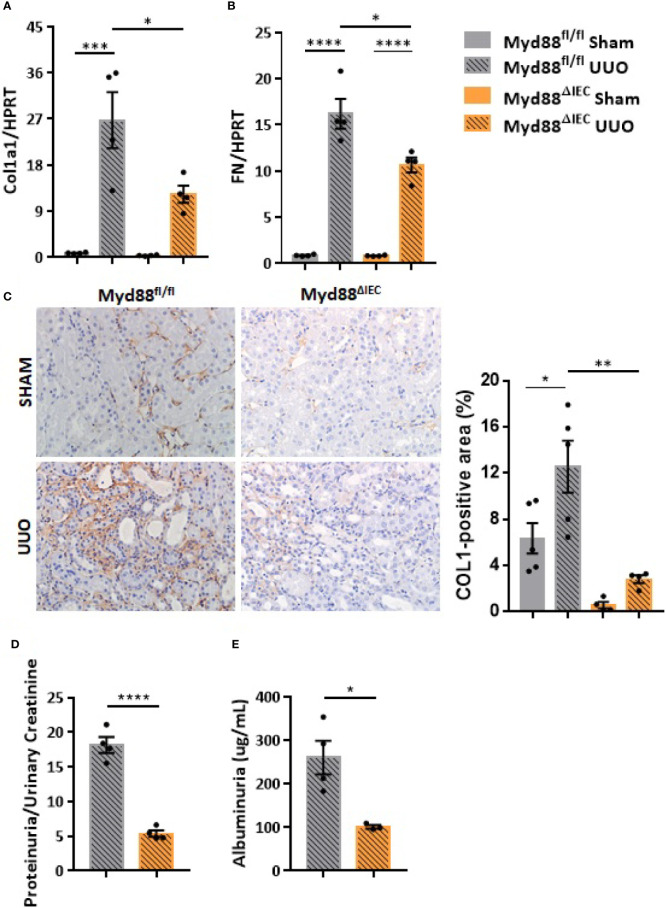
Effect of Myd88 signaling in IECs on the kidney remodeling in CKD. Gene expression of **(A)** Col1a1 and **(B)** fibronectin (FN) in the kidney of Myd88^fl/fl^ and Myd88^ΔIEC^ mice submitted or not to UUO (N = 4). **(C)** Representative images and positive area (%) of immunohistochemical staining for type I collagen (Col I) in the kidney of Myd88^fl/fl^ (N = 5) and Myd88^ΔIEC^ mice (N = 4) submitted or not to UUO. **(D)** Concentration of total protein normalized by creatinine in pelvic urine (N = 4). **(E)** Estimation of albuminuria in pelvic urine collected from Myd88^fl/fl^ (N = 4) and Myd88^ΔIEC^ mice (N = 3) submitted to UUO. *p < 0.05; **p < 0.01; ***p < 0.001; ****p < 0.0001.

On the next step of the study, we decided to examine whether this UUO-generated inflammation would be dependent on Myd88 signaling in IECs. For this purpose, after confirmation of Myd88 deletion in IEC isolated from intestine (**Figure S2**), Myd88^ΔIEC^ mice were submitted to UUO and expression of proinflammatory cytokines was evaluated aiming to verify the participation of IEC-specific Myd88 signaling in local and systemic inflammation. Analysis of colon samples of Myd88^ΔIEC^ mice, similar to untreated WT mice, revealed that UUO led to a massive bacterial invasion in the mucus layer (**Figure 4A**). Surprisingly, a higher bacterial burden was only observed in MLN of untreated WT mice after UUO (**Figure 4B**). In addition, the lack of Myd88 in IECs decreased levels of IL-1b, IL-12p40, and IL-17A in the colon after UUO, while levels of TNF-α and colony stimulating factors, such as GM-CSF and MIP-1β, were unaltered in both strains (**Figure 5**). Kidney assessment revealed that Myd88^ΔIEC^ mice submitted to UUO presented lower levels of IL-4, IL-12p40, IL-17A, GM-CSF, and MIP-1β compared to Myd88^fl/fl^ mice (**Figure 6**). Besides the attenuation of the proinflammatory scenario, the selective deletion of Myd88 also influenced the expression of profibrotic markers. Myd88^ΔIEC^ mice submitted to UUO presented lower expression of Col1a1 and fibronectin compared to Myd88^flox^ mice followed by decreased deposition of type I collagen (**Figure 7**). Renal benefits also included the observation of better pelvic urinary parameters (**Figure 7**). Low urinary levels of total protein and albumin were observed in Myd88^ΔIEC^ mice.

## Discussion

In the last decades, important advances have shown the essential role of gut microbiota in host physiology, including modulation of host metabolism, central nervous system activity and, especially, immune system. Several studies have shown that gut microbiota may dictate how host immune system would respond to inflammatory hits (17–19). Despite the increasing data supporting the association of CKD and dysbiosis, little is known whether alteration in the gut microbiota would influence the onset and development of CKD. Therefore, in this study we evaluated the effect of depletion of gut microbiota and the role of Myd88 signaling in IECs during UUO injury. Changes in the gut microbiota induced by ABX treatment were followed by upregulation of occludin and ZO-1, which are considered critical components of tight junctions. Substantial data have correlated the expression of both components with epithelial barrier properties. Suppression of occludin or ZO-1 in epithelial monolayer culture alters the dynamic behavior of tight junction by increasing leak pathway flux (20, 21). Moreover, overexpression of occludin limited intestinal barrier disruption in an experimental TNF-induced diarrhea (22). Despite our results may be an indicative of a pre-reinforcement of tight junctions, there is not a consensus whether ABX treatment impairs the intestinal barrier integrity. Tulstrup et al. did not observe alterations in the gene expression of ZO-1, occludin, claudin-1 suggesting that ABX effect is dependent on ABX class (23).

Depletion of gut microbiota abrogated inflammation induced by UUO in the colon. The presence of gut microbiota seems to be crucial to trigger local UUO-induced inflammation and intestinal barrier disruption. Untreated mice showed high expression of proinflammatory cytokines, such as IL-1b and IL-6, in the colon along with a massive presence of bacteria in the mucus layer. This bacterial burden was not limited to the mucus layer since we detected a higher bacterial load in the MLN after UUO. In physiological conditions, the intestinal immune system is taught by the gut microbiota to tolerate luminal antigens, thus limiting host immune responses. However, in dysbiosis conditions, such as the one generated by UUO, a higher trafficking of microbial antigens *via* CX3CR1^hi^ mononuclear phagocytes can occur aiming at compartmentalizing the intestinal immune response to avoid inflammation (24). Indeed, we did not detect alterations in bacterial load in the liver after UUO, which supports the idea of MLN as a key immune inductive site. Taken together, these observations indicate that depletion of gut microbiota protects against UUO intestinal injury probably by lowering the load of bacteria, which could play a pathogenic role when the intestinal barrier is disrupted. This scenario is aggravated by increased serum levels of IL-6, which support the idea of a sustained inflammatory status generated by CKD.

Beneficial effects of depleted gut microbiota reach the kidney reinforcing the importance of kidney-gut axis in CKD. ABX-treated mice presented lower expression of proinflammatory cytokines after UUO compared to untreated mice. Moreover, depletion of gut microbiota downregulated the expression of several profibrotic markers, such as type I collagen, fibronectin and α-SMA, alleviating tubulointerstitial fibrosis, which is a main histological hallmark of CKD. Moreover, ABX treatment also reduced the expression of cell activation markers, such as FSP-1 and KI-67. During tubular injury, increased FSP-1 expression allows the tracking of cellular behavior associated with matrix production, including cell phenotypic transition, migration, and invasiveness. Evidences indicate that the origin of kidney fibroblasts includes perivascular and resident fibroblasts, pericytes (25), bone marrow derived fibrocytes (26), and transformed epithelial cells (27). Increased expression of KI-67 as observed in untreated mice is an indicative of proliferation of tubular epithelial cells and fibroblasts. All the aforementioned renal improvement fostered the preservation of kidney structure highlighting the idea that, despite the benefits generated by gut microbiota in physiological conditions, the bacterial community can feed inflammatory states generated by other hits.

The importance of IECs in maintenance of intestinal barrier is clearly highlighted in infectious diseases and pathologies with host immune genetic susceptibility. Of great relevance to intestinal homeostasis, Myd88 is a crucial player in the interface between host and microbes and essential to host resistance to infection (28, 29). Deletion of Myd88 decreases AMP secretion, such as RegIIIγ (30) and RELMβ (28) and impairs epithelial cell turnover and repair (31) leading to intestinal barrier dysfunction in experimental infectious colitis. Therefore, in the next step of the study, we decided to evaluate the impact of Myd88 signaling in IECs in the onset and development of CKD. The lack of Myd88 signaling led to lower levels of cytokines in the colon, such as IL-1b, IL-12p40, and IL-17A, in the CKD condition. This significant reduction indicates that, at least locally, Myd88 signaling is essential to the production of critical cytokines involved in the maintenance of intestinal barrier, probably by decreased signaling after PAMP sensing. Unlike in other organs, in the colon IL-1b has a differential role and appears to influence epithelial proliferation promoting healing and tissue repair (32). IL-17A also promotes epithelial barrier integrity by regulating the cellular localization of occludin (33) and regulates antimicrobial activity at mucosal surface (34). On the other hand, IL-17-producing cells can be pathogenic in intestinal diseases, when co-expressed with IL-23, demonstrating a dual role for this cytokine in gut homeostasis and disease (35). Despite the downregulation of these critical cytokines, we did not observe differences in the pattern of bacterial invasion in the mucus layer between WT and Myd88^ΔIEC^ mice. Apparently, activation of myeloid cells *via* Myd88 is necessary for the development of chronic intestinal inflammation, whereas epithelial Myd88 signaling is essential for host survival (36). This cell-specific role of Myd88 could explain why we did not observe any evidence of intestinal inflammation in the scenario of IEC-deletion of Myd88. On the other hand, unexpectedly, the lack of Myd88 signaling in IECs prevented a bacterial burden in MLN after UUO, indicating that TLR-mediated epithelial recognition of luminal bacteria is required for bacterial trafficking, probably *via* CX3CR1^hi^ mononuclear phagocytes, aiming at induction of local immune response to avoid systemic inflammation (24).

The lack of Myd88 in IECs also modulated inflammation in the kidney supporting the importance of kidney-gut axis in CKD. Myd88^ΔIEC^ mice presented lower levels of proinflammatory cytokines including IL-4, IL-12p40, and IL-17A, and reduced renal concentration of macrophage-stimulating factors, such as GM-CSF and MIP-1β. GM-CSF has been implicated in the crosstalk between cell tubular injury and cell immune infiltration involved in the sustained tubular injury and progressive interstitial fibrosis at the time of AKI-to-CKD transition (37). Indeed, the lack of Myd88 in IECs modulated the gene expression of profibrotic markers and abrogated the increase of type I collagen after UUO, indicating a substantial prevention of tubulointerstitial fibrosis. The attenuation of inflammation and fibrosis reflected in urinary parameters, once we observed lower levels of protein and albumin in pelvic urine of Myd88^ΔIEC^ mice, supporting the idea that Myd88 signaling in IECs participates in UUO injury. Here, we found that IEC-specific Myd88 deletion alleviated kidney injury indicating that, besides gut microbiota, innate signaling plays an important role in the microbiota-gut-kidney axis in CKD. IECs are capable to sense the microbial presence through a variety of antigen receptors expressed on their apical surface, leading to activation of innate and adaptative immune responses (38). Therefore, we can speculate that the beneficial effects of Myd88 deletion after UUO may be due to an attenuation of intestinal inflammation similarly observed in a model of intestinal injury induced by ischemia/reperfusion. Mühlbauer et al. reported that impaired epithelial Myd88 signaling decreased neutrophil infiltration, reduced binding of IgA to neoantingens, as well as diminished complement activation (39). Myd88 signaling in IECs may affect the maturation of renal resident macrophages and of bone marrow monocytes recruited during inflammatory conditions. Indeed, Emal et al. reported the critical role of gut microbiota in keeping maturation/supply status of renal macrophages in an ischemia-reperfusion injury (40), supporting the importance of gut microbiota-host interaction in modulating the systemic immune system and ultimately inflammatory conditions. Several studies, especially using germ-free mice, demonstrated the importance of microbiota and host interactions in priming immunity (19, 41, 42), which could have deleterious effects in inflammatory states, such as kidney injury.

In summary, we showed that depletion of gut microbiota protects against UUO injury supporting the idea that gut microbiota and host interactions can be detrimental in inflammatory pathologies, such as CKD. Moreover, the renal benefits generated by Myd88 deletion in IECs indicate that the deleterious effects of gut microbiota in such conditions may be dependent of host innate signaling.

## Data Availability Statement

The original contributions presented in the study are included in the article/**Supplementary Material**. Further inquiries can be directed to the corresponding author.

## Ethics Statement

The animal study was reviewed and approved by Ethics Committee in Animal Research of the Federal University of São Paulo.

## Author Contributions

IW and NC conceived and initiated the study. IW, MA-S, OF-N, and NC planned the experiments. IW, MA-S, OF-N, and RF performed the experiments. MM, RC, MC, TH, LP, and RV gave technical assistance. IW, MA-S, OF-N, and NC analyzed the data. IW, AP-S, and NC wrote and revised the manuscript. All authors contributed to the article and approved the submitted version.

## Funding

This work was supported by Fundação de Amparo à Pesquisa do Estado de São Paulo (FAPESP, São Paulo, Brazil) (grant numbers 2014/50833-1 and 2017/05264-7), Conselho Nacional de Desenvolvimento Científico e Tecnológico (CNPq) and Coordenação de Aperfeiçoamento de Pessoal de Nível Superior (CAPES, financial code 001). This work was also supported under the International Collaboration Research Funding from FAPESP and The Netherlands Organization for Scientific Research (NWO, The Netherlands, Grant number 2019/19435-3). This work was also a result of collaboration between NC and Ivan Cruz Moura at Institute Imagine, Paris, under the support of CAPES Cofecub Program (Grant number 19/954).

## Conflict of Interest

The authors declare that the research was conducted in the absence of any commercial or financial relationships that could be construed as a potential conflict of interest.
