# Supplementary material for: Gut Microbiota and Intestinal Epithelial Myd88 Signaling Are Crucial for Renal Injury in UUO Mice
Source: Front Immunol. 2020 Dec 22;11:578623. doi: 10.3389/fimmu.2020.578623 (PMC7783078; doi:10.3389/fimmu.2020.578623)
Supplement: Supplementary file 1 [file Presentation_1.pptx]

## Slide 1
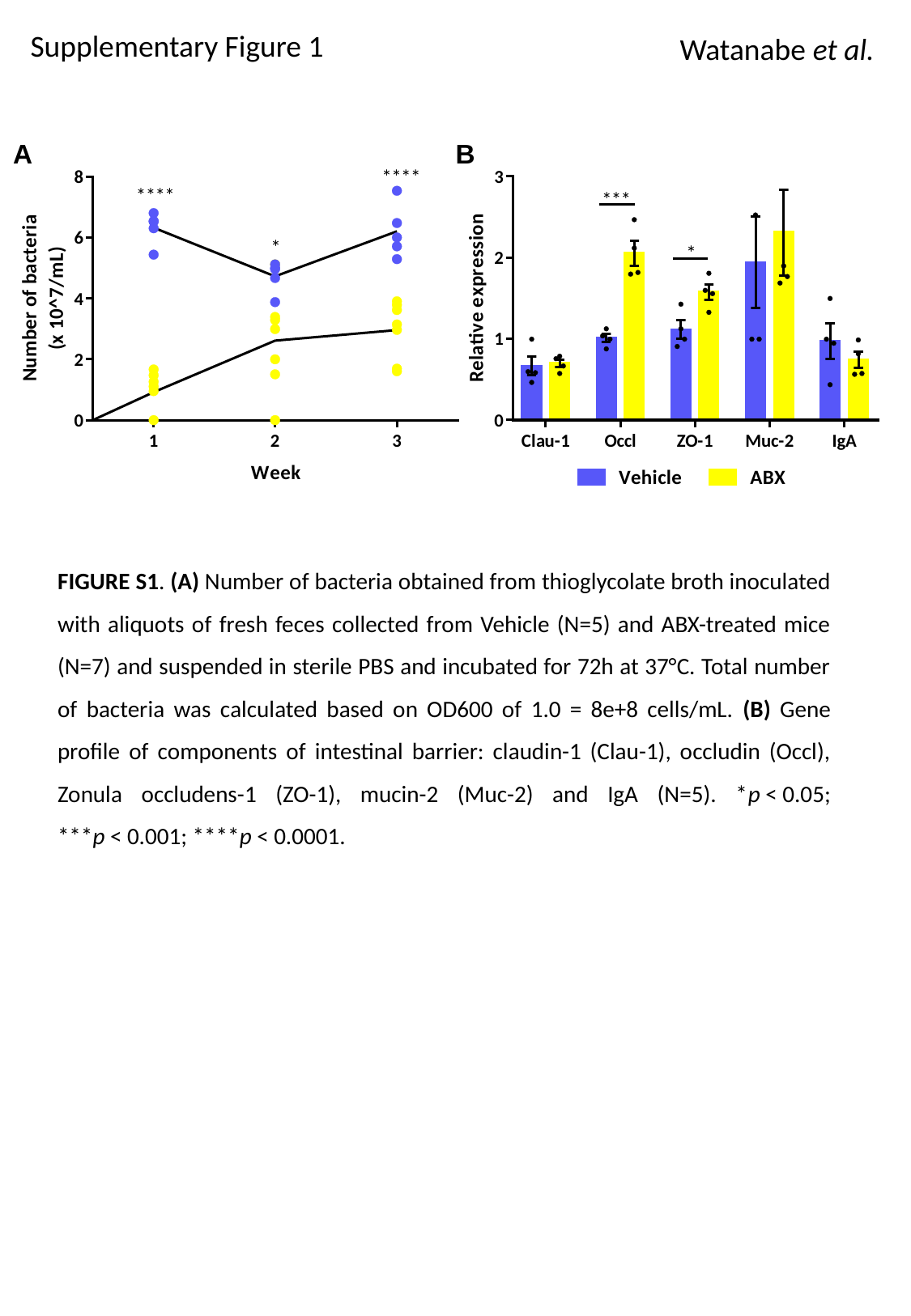

Supplementary Figure 1
Watanabe et al.
A
B
Figure S1. (A) Number of bacteria obtained from thioglycolate broth inoculated with aliquots of fresh feces collected from Vehicle (N=5) and ABX-treated mice (N=7) and suspended in sterile PBS and incubated for 72h at 37°C. Total number of bacteria was calculated based on OD600 of 1.0 = 8e+8 cells/mL. (B) Gene profile of components of intestinal barrier: claudin-1 (Clau-1), occludin (Occl), Zonula occludens-1 (ZO-1), mucin-2 (Muc-2) and IgA (N=5). *p < 0.05; ***p < 0.001; ****p < 0.0001.

## Slide 2
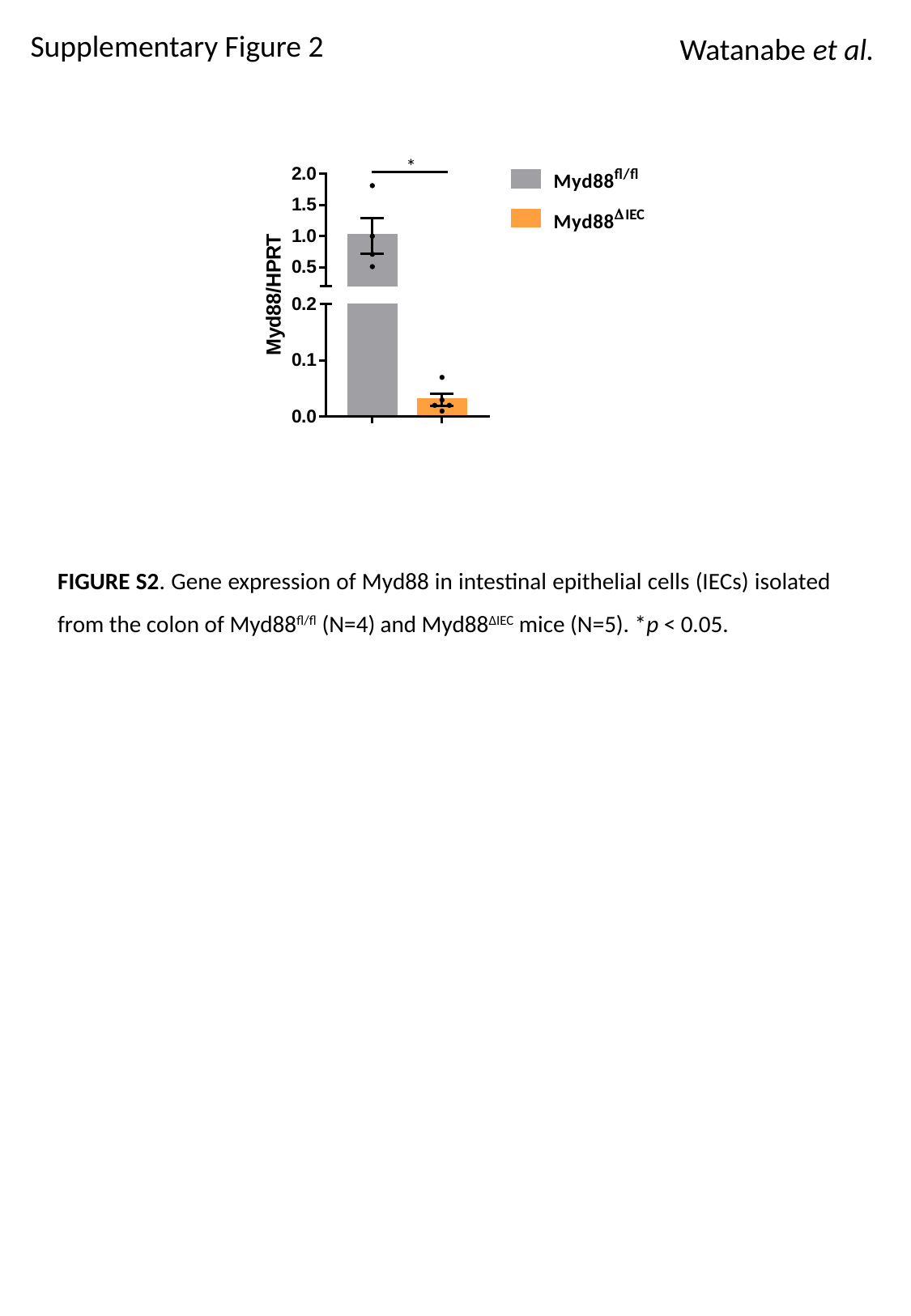

Supplementary Figure 2
Watanabe et al.
Figure S2. Gene expression of Myd88 in intestinal epithelial cells (IECs) isolated from the colon of Myd88fl/fl (N=4) and Myd88ΔIEC mice (N=5). *p < 0.05.
